# A systematic review of the impact of future-oriented thinking on academic outcomes

**DOI:** 10.3389/fpsyg.2023.1190546

**Published:** 2023-06-19

**Authors:** Simon Pawlak, Ahmed A. Moustafa

**Affiliations:** ^1^School of Psychology, Faculty of Society and Design, Bond University, Gold Coast, QLD, Australia; ^2^Department of Human Anatomy and Physiology, the Faculty of Health Sciences, University of Johannesburg, Johannesburg, South Africa; ^3^Centre for Data Analytics, Bond University, Gold Coast, QLD, Australia

**Keywords:** academic outcomes, engagement, episodic prospection, future-oriented thought, mental contrasting, prospection, motivation, time perspective

## Abstract

Future-oriented thought is a broad construct that characterize the ability to generate mental representations of the future and project oneself into a variety of hypothetical states. It is well established that the degree to which one is focused more on the past, present, or future has a variety of implications on psychological functioning. This study focuses on the relationship between future-oriented thought and academic performance of students. To bridge this gap, we conducted the first systematic review investigating the benefit of future-oriented thought on promoting positive outcomes in academic settings. Our systematic review comprised 21 studies (*k* = 21). Results identified a clear relationship between future-oriented thought and positive outcomes in academic settings. Furthermore, our systematic review reveals important relationships between future-oriented thought and academic engagement, as well as future-oriented thought and academic performance. Our findings suggest that those who are more future-oriented demonstrate higher levels of academic engagement compared to those who were less future-oriented. Our findings suggest that probing and guiding students toward a future goal may increase their academic engagement and performance.

## Introduction

Future-oriented thinking is a broad construct, which characterizes the various cognitive capabilities employed to generate future states of thought and project oneself into a variety of hypothetical scenarios in the future (Atance and O'Neill, 2005; Szpunar et al., 2014). The ability to mentally project oneself into a desired future state—whether that be a mental simulation as a keynote speaker or achieving a high distinction on a university assignment is argued to be an important function in our capacities as human beings to set goals and guide behavior (Stanescu and Iorga, 2015; Andre et al., 2018; Berkman, 2018).

Approaching goals with the future in mind has been found to increase the perceived likelihood of goal-attainment and achievement. Szpunar and Schacter (2013) demonstrated that repeated mental simulations of a future event increase individual judgments toward the perceived likelihood that the event will take place. Importantly, Oettinger and Mayer (2002) identified that an increase in the belief that a goal will be attained has been found to be positively correlated with engagement and self-efficacy; factors that have been consistently implicated in fostering positive academic outcomes (Gerber et al., 2013; Dogan, 2015; Delfino, 2019; Foster et al., 2019). Millions of people around the world will either begin or end their day setting a goal, or to some degree, envisioning what success looks like to them. Given that goal attainment is situated in the future, and that positive outcomes have been identified when approaching tasks with the future in mind (Ernst et al., 2018), future-oriented thinking is an important construct to unpack in greater depth.

There are two perspectives from which goal achievement can be understood: (1) short-term (e.g., “this week I am going to study for 2 h per day”), and (2) long-term (e.g., “in 5 years' time I will be a registered psychologist”). In this context, the structure of the goal, that is, short- or long-term, is referred to as *temporal distance*, which is the time between the present-self and target event (Stein et al., 2018). Temporal distance is important when considering the likelihood of goal self-congruence, which refers to the degree to which one actively works toward achieving a goal, which is consistent with the view that one holds of themselves (Peetz et al., 2009; Ernst et al., 2018). It is argued that short-term goals are often easier to establish than long-term goals, as short-term goals require less cognitive input, such as implementation and planning facilitated by the prefrontal cortex (Berkman, 2018), are more concrete (i.e., grounded in the present), and are considerable drivers of self-regulatory behavior, such that they contain more instant motivational properties (i.e., gratification and reward) that can be accessed earlier (Bulley et al., 2016).

However, long-term goals are situated at a greater temporal distance, and thus typically more abstract—often requiring greater effort to sustain goal-directed behaviors as the reward of achievement is delayed (Bulley et al., 2016). Other factors impacting long-term goals include goal self-congruence (Ernst et al., 2018), implementation planning (Carraro and Gaudreau, 2011), and other psychosocial factors, such as motivation and self-efficacy (Tindle et al., 2021). Self-concordant goals (i.e., goals that are aligned with personal values) are argued to be more effective than goals that are non-self-concordant, due to the expectation that they are more likely to satisfy psychological needs (Ernst et al., 2018). Conversely, the degree to which a goal is congruent with an individuals' desired future state is critical when considering engagement in the steps required to achieve a planned goal (O'Donnell et al., 2017).

With respect to goal achievement, previous work has explored the relationship between goal-directed behaviors (such as engagement and planning) and future-oriented thought in a variety of settings, such as the workplace, health care, and academia. Demonstrating that the degree to which an individual is more oriented toward the future and less oriented toward the past yields an array of positive outcomes (Andre et al., 2018; Crone et al., 2020; Coughlin et al., 2022). It is argued that future-oriented thought is beneficial within contexts that require the individual to demonstrate behaviors that are congruent with intended outcomes (Hoyle and Sherrill, 2006). An example is engagement in academic settings, which Rissanen (2018) identified to be a critical factor in promoting positive outcomes (high grade-point average). Future-oriented thought is argued to promote several adaptive functions that elicit engagement and motivation in a variety of contexts. With respect to goal pursuit, Duckworth et al. (2013) argued that future-oriented thought provides an enhanced capability for strategic planning and acts as a protective factor against goal-disengagement (giving up). Through visualizing obstacles that may arise when pursuing a goal, the individual can implement solutions to overcome any obstacles that may arise.

Markus and Nurius (1986) argued that the adaptive function of future-oriented thought is that it promotes motivation. They identified that through visualizing an optimal version of the self (the best possible version oneself can become) and contrasting this vision with a feared self (a self that one wishes never to become), the individual demonstrates an increase in effort and engagement toward tasks that contribute to the creation of the optimal future self. Considering what has just been discussed, the primary goal of this study is to assess whether future-oriented thinking is advantageous in eliciting positive outcomes within an academic setting. Importantly, because there is a range of terminology in which researchers refer to when discussing future-oriented thought, we think it is important to present and describe these prior to unpacking future-oriented thought in further depth. As such, an overview of future-oriented thought terminology and construct definitions is provided below see Table 1.

**Table 1:** Future-Oriented Thought: Constructs and Terminology.

**Author/s**	**Construct**	**Description**
Lewin (1942)	Time perspective	A cognitive bias toward a particular temporal state – i.e., a bias to be fixated on the past, present or future
Nurmi (1991)	Future time perspective	An individual's attitude toward the future
Duckworth et al. (2013)	Mental contrasting	A self-regulatory strategy for goal-pursuit situated in the future
Markus and Nurius (1986)	Possible selves	A future-oriented self-concept; characterized by a hoped for, feared, or ultimate self
Strathman et al. (1994)	Future consequences	The tendency of an individual to take into account the future consequences of his/her behavior
Kappes et al. (2012)	Positive fantasies	Fantasies that are future-oriented, which depict the future as bright and easily attained
Mello and Worrell (2006)	Time attitude	Positive and negative attitudes which are oriented toward the past, present, or future
Bartels and Urminsky (2011)	Future Self-Continuity	The degree to which the individual feels connected with, and harmonious with their desired future self
McGuire (1990)	Psychological Anticipation	A conceptual amalgamation of thoughts, beliefs, and knowledge of the future to construct a proposition or evaluation as to the probability of a future outcome
Ruvolo and Markus (1992)	Possible Identities	A mental representation of something, or someone an individual can become

Though various terminologies are utilized to characterize future-oriented thought as discussed above, all of them prescribe the ability to: (1) generate a mental representation of a desired future state; (2) anticipate the likelihood of achieving desired future state; (3) construct a goal to achieve desired future state; (4) organize oneself to identify the steps needed in order to achieve desired future state. All of which are argued to be adaptive functions of future-oriented thought (Schacter et al., 2008, 2017; Szpunar and McDermott, 2008; Anderson and Evans, 2015; Moustafa et al., 2018; Hollis-Hansen et al., 2019; Moustafa and Morris, 2019).

When understanding the application of future-oriented thought in an experimental setting, such as investigating the utility of the construct in a motivational capacity, the literature suggests that future-oriented thought is utilized in two beneficial ways. First, it is utilized as a measure of orientation, that is, the degree to which one is focused on the past, present or future. For example, the Zimbardo Time Perspective Inventory (1999) is a widely used inventory that assesses individual experiences of time. This inventory has been utilized in research conducted by Adelabu (2008) in effort to understand how individual tendencies to be more past, present, or future-focused impacts college grade-point average (GPA). Second, future-oriented thought can be delivered as an intervention, such as, Mental Contrasting with Implementation Intentions (MCII), which is developed by Kirk et al. (2012). The MCII seeks to enhance an individual's goal-setting capabilities through visualizing an optimal future state, considering the steps that are required to be taken to reach this state whilst contrasting obstacles that may be presented along the way. For a more detailed overview of interventions and inventories see Table 2.

**Table 2:** Future-Oriented Thought: Inventories and Interventions.

**Exercise/Inventory**	**Author**	**Type**	**Description**	**Item example**
Zimbardo Time Perspective Inventory, ZTPI	Zimbardo and Boyd (1999)	Inventory	Widely used questionnaire designed to measure experiential dimensions of time – such as tendency to focus on the past, present, or future	“I am able to resist temptations when I know there is work to be done.”
Mental Contrasting with Implementation Intentions, MCII	Kirk et al. (2012)	Exercise/intervention	Facilitates the conversion of thoughts about desired futures into goals; Implementation intentions link obstacles to specific actions that overcome them	“i.e., *if* obstacle, *then* goal-directed action”
Consideration of Future Consequences Scale	Strathman et al. (1994)	Inventory	The scale measures the extent to which people consider the future and immediate consequences of their behaviors	“I consider how things might be in the future, and try to influence those things with my day-to-day behavior.”
Possible Selves	Oyserman and James (2012)	Exercise/Intervention	An Intervention in which participants write down about themselves in their best possible future, and assess the likelihood of attaining such future	“What is the likelihood that you will get a good GPA?” Participants responded on a scale ranging from 1 = very unlikely to 7 = very likely.
The Adolescent Time Inventory	Mello et al. (2008)	Inventory	A 30-item scale used to measure adolescents' attitudes toward the past, present, and future	“Overall, I feel happy about what I am doing right now,”/(“I wish that I did not have the past that I had,”
Future Time Perspective Connectedness Scale	Husman and Shell (2008)	Inventory	A 16-item inventory that assesses the contingent or instrumental connection between current behavior and future goal achievement	“What will happen in the future is an important consideration in deciding what action to take now”

With consideration to the broad nature of constructs relating to future-oriented thought, it is important to convey key inventories and interventions more which are widely recognized in this field. As presented in Table 2, and discussed regularly throughout this paper, the utility of the above-mentioned inventories is utilized to establish baselines as to the degree to which one is future-oriented. Examples include the Zimbardo Time Perspective Inventory (Zimbardo and Boyd, 1999). Furthermore, the interventions discussed provide the individual with the necessary capability to enhance the degree to which one is future-oriented. Examples include the Possible Selves Intervention (Oyserman and James, 2012).

### An Argument for Relevance

An important context in which future-oriented thinking is argued to be beneficial is that of academic settings, specifically, middle-school, high-school and higher education such as university (Bowles, 2008; Rieckmann, 2012; Landau et al., 2014, 2017). Although the motivational function of future-oriented thinking has been explored in other settings such as the workplace (Gupta et al., 2012) and in health (Cross and Sheffield, 2019), the academic setting is a unique context, in that not only are students navigating the complexities of identity exploration in mid-late teenage years through to emerging adulthood, but it is also a time that is sensitive for career consideration and pursuit (Arnett et al., 2014; Shulman et al., 2015). As participation to learn in an academic environment is self-directed, factors such as attitude (Madrazo, 2021), choice of best study strategy (De Beni and Moè, 2003), engagement (Chishima and Wilson, 2021), self-esteem (Kinik and Odaci, 2020) and self-regulation (Clark et al., 2021) are of considerable importance to when considering the practical implications of future-oriented thought in the academic context.

### Correlates of academic performance

Within the academic context, Tindle et al. (2021) found psychological well-being to be significantly correlated to with academic performance. Specifically, it was identified as a critical factor in fostering self-efficacy and motivation. According to findings from the Lamb et al. (2020), positive outcomes in academic settings are linked to several important psychosocial factors. Namely, a sense of purpose and self-efficacy. These factors have not only been identified to be an interpersonal competency of the student, however reciprocal to student-teacher interactions in both high-school and university settings (Hagenauer et al., 2015; Kim et al., 2018; Li and Yang, 2021). These findings are corroborated by Robbins et al. (2004), who conducted a meta-analysis of 109 studies that investigated psychosocial predictors of academic performance. A student's belief in their academic ability was identified as the most powerful predictor of positive outcomes.

Furthermore, key statistics from the Education Opportunity in Australia Report (2015) highlight that 40.5% of male and 41.4% of female high-school students reported lower levels of self-efficacy compared to same aged peers. With consideration to the aforementioned, it is worthwhile considering the current efforts within the field of future-oriented thinking and contrasting findings in order to assess the efficacy of future-oriented thinking within an academic context.

### Future-oriented findings

Most of the research that have taken place over the last twenty years in this field have explored the utility of future-oriented thinking with an emphasis on goal-directed behaviors, investigating contexts in which future-oriented thinking can enhance self-efficacy (McMichael et al., 2022), increase individual engagement (Gupta and Bakker, 2020), as well as establish goal self-congruence (Henry, 2020). The literature suggests that up until this point, “Possible Selves” or “Best Possible Selves” is the most widely researched future-oriented cognitive phenomena within an academic context. Though similar, “Possible Selves” or “Best Possible Selves” differ from an intervention based perspective. In other words, Possible Selves, developed by Markus and Nurius (1986) characterize the mental representations that one can create which demonstrate their ultimate, most successful and hoped for self, or their most feared self. However, Best Possible Selves is delivered as an intervention. Developed by King (2001), it is an activity in which participants are instructed to write about themselves in the future, imaging that everything has worked out in the best possible way.

Several key aspects remain consistently discussed and interest researchers when investigating the motivational properties of future-oriented thought. These are (1) *salience* (how vivid mental representations of the future are); (2) *relevance* (does the mental representation contribute to an outcome that is personally meaningful to the individual); (3) *organization* (does the mental representation contain fundamental aspects of goal pursuit, such as planning and preparation) (Nurmi, 1991). For example, the salience of the future was measured in a longitudinal study by McMichael et al. (2022) who explored changes in how vivid college students perceived their future over time, specifically at the start, and end of their degree, and the impacts that changes in future vividness had on academic performance. It was identified that those who maintained a clear vision of what their college graduation, and life after graduation would look like demonstrated stronger belief in their academic capabilities, in turn, leading to a better overall GPA, when compared to those who displayed less future vividness.

Further, Oyserman et al. (2006) explored the plausibility of possible selves impelling goal-directed behavior within a classroom setting; finding that unless linked with realistic strategies, visualizing an academically successful possible self is not enough to elicit behaviors congruent with an ideal self. It was identified that those who implemented strategies in line with their ideal academic self (such as class preparation and study) resulted in improved academic initiative and test scores. Interestingly, a subsequent yet unexpected finding that emerged from the experimental group was in relation to class absence. When compared with a control group, absence from school reduced when compared with baseline attendance.

### Future-orientated thinking as a mechanism for motivation

When in pursuit of a goal, or in circumstances where positive outcomes are determined by the contribution of individual effort—that is, how well one performs on a university assignment, or overall Grade Point Average (GPA) upon graduation, is often determined by the level of engagement, and how aligned the individuals' behaviors are to the intended outcome (Miller and Brickman, 2004). The academic context in this instance presents an important opportunity to discuss the relevance of future-oriented thought in relation to goal-directed behavior and self-congruence with respect to degree attainment. A unique aspect of pursuing a degree in academia, whether that be an undergraduate, post-graduate diploma or finishing high school is recognized in delays of reward or gratification (also known as delayed gratification). Being recognized as a key contributor to sustained effort, Berkman (2018) suggests that reward and recognition are not typically realized in academia for years due to the typical length of university degrees. In Australia, this can be upwards of four years for an undergraduate degree (Study Australia, 2022). Thus, stagnation in effort and performance, or withdrawal from study is common, especially amongst 18–25 year old individuals. This is attested by recent research investigating drop-out rates in Australia, which indicated that 1 in 5 undergraduate students drop out of university (Shipley and Ian, 2019; Lamb et al., 2020).

With this in mind, critical voices have pursued a more in-depth understanding of the behavioral and motivational impact of future-oriented thought in academia specifically, and across several life domains including tertiary settings, health, and the workplace. For example, when examining visualizations of possible future selves on self-efficacy and engagement in university students, those who maintained a representation of a successful future demonstrated an increase in self-efficacy and engagement when compared with those who maintained a representation of a general future failure toward their degree (de Place and Brunot, 2020). Along these lines, Andre et al. (2018) explored the relationship between motivation and future-oriented thought across education, the workplace, and in health related outcomes. When examining the difference between those who demonstrated a domain specific future-time orientation with those who express more general future-time orientations, significant differences in behavioral outcomes were identified. Specifically, with respect to the educational domain, those who demonstrated specific future-time orientations (a specific outcome or goal related to their degree) when compared those who demonstrated general future-time orientations (non-specific outcomes) exerted greater effort and course work performance (Andre et al., 2018).

Moreover, the importance of the socio-cultural context in student motivation and achievement has been demonstrated by Worrell et al. (2021). They have highlighted that future-orientation acts as a protective factor to students raised in households of neglect and deprivation. Future-orientation was identified to provide a robust foundation of self-efficacy when considering attitudes toward the future, supporting motivation to study. The socio-cultural context has been implicated in previous works conducted by Engin (2020), who undertook a descriptive assessment of a cohort of 60 students. Findings demonstrated that students who were raised in households with fathers who attained a high level of education exhibited more engagement and higher self-efficacy.

### The current study

Although future-oriented thinking has been positively implicated in academic outcomes (Oyserman et al., 2006, 2015; de Place and Brunot, 2020; Chishima and Wilson, 2021), to what degree more broadly future-oriented thinking facilitates positive outcomes in academia remains to be investigated. In other words, a broad consensus has yet to be reached as to the functional benefit of future-oriented thinking, and the impact that future-oriented thinking has on positive outcomes such as engagement and performance (GPA) within an academic setting. Moreover, findings in this field remain disconnected, such that, to date only a handful of studies have sought to integrate prior findings (Andre et al., 2018; Loveday et al., 2018). To the best of our knowledge, no reviews have attempted to synthesize literature on future-oriented thinking in relation to positive outcomes more broadly within an academic setting. As such, this systematic review will, for the first time, seek to combine and contrast the findings of future-oriented thinking in relation to positive outcomes in an academic setting. In doing so, we posit the following question: *Does future-oriented thinking promote positive outcomes in an academic setting?* In the hopes of endorsing the utility of future-focused practices in academia, and goal attainment more broadly.

## Method

### Literature search, study selection and inclusion criteria

Comprehensive literature search procedures were undertaken on all major databases such as PsychINFO and EBSCOhost, utilizing a combination of search terms such as “*future thought AND academic outcomes*” or “*engagement AND possible selves AND academic outcomes AND academic performance*.” Literature search parameters required the search to: (1) yield articles published after the year 2000; (2) were written in English; (3) were peer reviewed; (4) were not books, theses, or book chapters. Searches were expanded to include articles on Google Scholar as well as backward citation searching through included texts for any articles that may have been missed in primary search.

### Study selection and inclusion criteria

The criteria for inclusion/exclusion for each stage were developed prior to beginning the initial search. A three-stage method was employed to determine eligibility based on specified inclusion/exclusion criteria at each stage. That is, *Title screen, Abstract screen, and Full-text screen*, with consideration to an immediate removal of duplicates prior to screening. Inclusion into this systematic review required studies to meet all of the following criteria: (a) the study be quantitative and not qualitative; (b) the dependant variable (DV) was academic outcome related; (c) a variation of future-oriented thought was an independent variable (IV); (d) at least one general measure of future-oriented thought is utilized, such as the Zimbardo Time Perspective Inventory (Zimbardo and Boyd, 1999), Consideration of Future Consequences Scale (Strathman et al., 1994), or Future Time Orientation Scale (Gjesme, 1975). Moreover, further consideration was provided to the time orientation of the future-oriented variable, such that, a future component must be included, and either a baseline measure or control group is present. As our research sought to identify studies that explored positive outcomes related to academia, the parameters for variables related to academic outcomes required a positive outcome. That is, an observable behavior such as class attendance, contribution, homework hours completed, initiative etc.; or an observable performance related measure, such as (GPA) that is favorable in an academic context.

**Figure 1:**
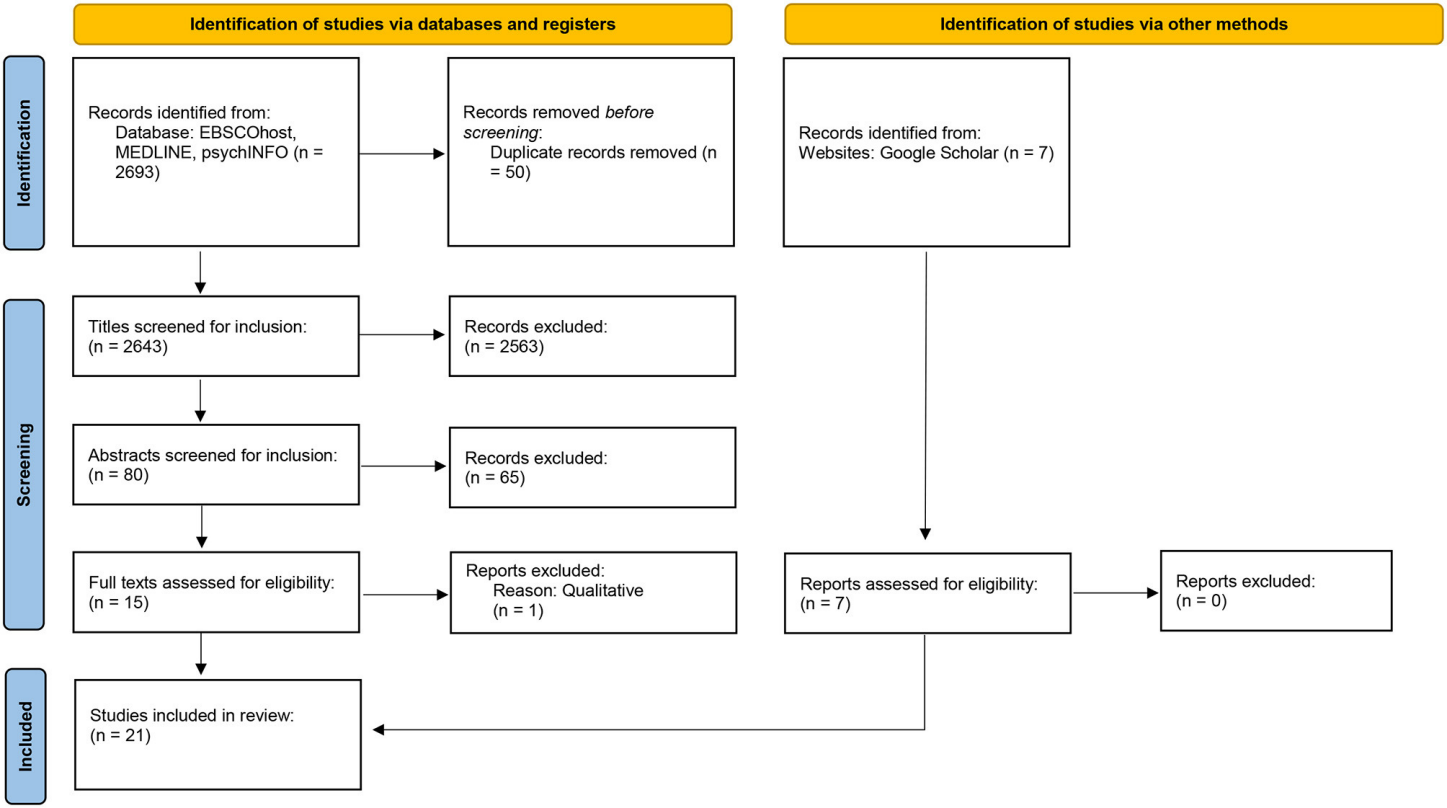
PRISMA Flow-process.

Further requirements were that the experiment was conducted on non-clinical samples, that is, mentally healthy individuals (e.g., students). Studies which considered clinical samples were excluded as symptoms may pose as extraneous variables, which may affect efficacy of the intervention potentially confounding results (Ewert and Sibthorp, 2009). With respect to all research articles, 2,699 were imported into EndNote Reference Manager. Upon importing them, 50 articles were excluded immediately due to being identified as duplicates; 2,649 article titles were scanned, 2,569 articles were excluded due to titles not matching criteria, exclusion was based on either not being an experiment of interest or simply demonstrating a one-keyword similarity to the search term. Of the 80 articles remaining, abstracts were scanned for consideration of a quantitative aspect; a future-oriented component; and a positive academic outcome, 66 articles were excluded due to not meeting criteria. Subsequently, 7 articles were identified via google scholar and included in the systematic review. Twenty articles remained for Full-text review—all 21 articles matched the pre-determined search criteria and were consequently included into this systematic review. The above-described process was informed by Moher et al. (2009) the preferred process for reporting items for Systematic Reviews and Meta-Analysis (PRISMA); a 27-item checklist utilized in order to improve transparency in systematic reviews (see Figure 1 for PRISMA flow-process).

## Results

### Synthesis of findings

The dependent variable required for inclusion was a measure of a positive academic outcome, such as attendance, engagement, or performance (GPA). Of the 21 included studies, 12 studies emphasized solely performance, such as outcome measures related to GPA or scorecard average. The remaining nine studies emphasized a blend of engagement and performance related outcomes, such as outcomes related to class attendance, participation, reduced absent days, as well as increased language abilities. Based on assessment of the papers included in the systematic review, key findings were grouped into two main categories, enabling readers to draw clear, concise, and meaningful insights as to the practical implications of findings. These categories are as follows: (1) inventories and academic outcomes, comprising 10 independent studies and (2) interventions and academic outcomes, comprising 11 independent studies. For a summary of key findings relating to inventories and academic outcomes see Table 3 below. Furthermore, for a summary of key findings relating to interventions and academic outcomes see Table 4.

**Table 3:** Future-oriented thought: inventories and academic outcomes.

**Author**	**Construct/Inventory**	**Outcome**	**Finding**
Adelman et al. (2017)	Future Self-Continuity	Academic Performance	Findings outline that future-oriented thought may have positive effects through altering temporal focus (attention) from present state to a more future-focused state resulting in a demonstration of higher self-control
Adelabu (2008)	Time Perspective	Academic Performance	Findings indicate that those who demonstrate greater future orientation academically outperformed those who are less future-oriented
Andretta et al. (2014)	Time Attitude Profiles	Academic Performance and Engagement	Adolescents who demonstrated a positive/optimistic future outlook performed better than adolescents who demonstrated a negative/pessimistic future outlook
Barnett et al. (2020)	Time Perspective	Academic Performance	Future time perspective was found to increase academic engagement and GPA in a sample of college students after one semester, but not two semesters. Findings may indicate that the distance between the self and academic goal may demotivate. Robust planning and goal settings aligned with a positive future self may circumvent this
Coughlin et al. (2022)	Future-Oriented Thought	Academic Performance	Semantic prospection was identified to be most beneficial when comparing mean mathematic grades of students. Authors suggest that greater temporal distancing from goal may contribute to this finding
Horstmanshof and Zimitat (2007)	Future Time Perspective	Academic Engagement	Future time perspective was a significant predictor of academic engagement and intentions toward study. Such that, those who future-focused demonstrated deeper approaches to learning
King (2016)	Time Perspective	Academic Performance	Findings indicate that engagement was strongly associated with academic performance. Positive future and past time perspectives were identified to moderate the relationship between engagement and dissatisfaction
Mazzetti et al. (2020)	Future-Oriented Thought	Academic Performance	Learning strategies were found to be more successful when students reported higher levels of academic self-efficacy and future orientation. Learning strategies positively influenced GPA
Peters et al. (2005)	Future Consequences	Academic Performance	General well-being and academic GPA was assessed utilizing the Consideration of Future Consequences Scale. Peters et al. (2005) identified that those who reported higher on the Consideration of Future Consequences Scale reported having regular sleep schedules, better quality nights of sleep and higher GPA than those who reported lower on the Consideration of Future Consequences Scale
Shell and Husman (2001)	Future Time Perspective	Academic Engagement	Results identified that having a greater future time perspective plays a pivotal role in motivating academic achievement

**Table 4:** Future-Oriented Thought: Interventions and Academic Outcomes.

**Author**	**Construct/Intervention**	**Outcome**	**Finding**
Barnett et al. (2019)	Possible Selves	Academic Performance and Engagement	Students were instructed to participate in a future possible selves exercise. Those who received a letter from an unsuccessful future possible self, demonstrated a decrease in outcome expectancies. Potentially causing learned helplessness. Findings suggest that engaging with positive future possible selves may lead to an increase in self-esteem/agency to attain a higher GPA
Chishima and Wilson (2021)	Future Self-Continuity	Academic Engagement	The authors demonstrate that engaging with a future self through a letter exchange, students increased their engagement in career and course planning
Clark et al. (2021)	Mental Contrasting	Academic Engagement	Academic application (study time) was assessed through mental contrasting and a control condition with a sample of Australian University students. Over four weeks, those in the mental contrasting condition significantly increased amount of hours studied per week when compared with control
Crone et al. (2020)	Psychological Anticipation	Academic Performance	Anticipation of a future success was investigated in order to identify if it yielded positive academic outcomes. Findings suggest that anticipating future successes increases engagement through intentions
Duckworth et al. (2013)	Mental Contrasting	Academic Performance	Primary school students instructed on how to engage in mental contrasting with implementation intentions demonstrated greater class engagement, higher report card grades and a reduction in class absences when compared with control condition
Gollwitzer et al. (2011)	Mental Contrasting	Academic Performance	Mental Contrasting was taught to a sample of US middle school children from low-income neighborhoods. After two weeks, students achieved higher grades when compared with baseline performance. Suggesting that mental contrasting in beneficial in eliciting positive academic outcomes
Kappes et al. (2012)	Positive Fantasies	Academic Performance	Kappes et al. investigated whether positive fantasies alone are enough to increase educational outcomes in vocational courses. Findings demonstrated that simply thinking positively about ones future resulted in poorer educational outcomes
Koul et al. (2017)	Possible Selves	Academic Engagement	Results indicated that students who held a future possible self that represented growth were more inclined to engage in mastery orientation
Landau et al. (2017)	Possible Identities	Academic Engagement	Results demonstrated that students who engaged in creating an academically successful possible future self, and creating metaphors associated with the future self, reported higher levels of academic engagement
Landau et al. (2014)	Possible Identities	Academic Engagement	Results demonstrated that students who engaged in creating an academically successful possible future self, and creating metaphors associated with the future self, reported higher levels of academic engagement

## Discussion

The aim of this systematic review was to critically assess and disseminate current efforts in this field with the intention of drawing relevant and actionable insights regarding the relationship between future-oriented thinking and academic performance. Accordingly, above Table 4 provides a succinct overview of the key findings from each of the 21 studies included in this review. What can be drawn from the above findings are critical indicators that emphasize the efficacy of future-oriented thought as a competency that can be developed and employed in a variety of manners. In line with expectations, there is a considerable evidence that suggests exploring alternative methods of engagement such as future-oriented thought can increase the likelihood of positive academic outcomes in primary, secondary and tertiary settings.

Although research in this context has produced positive findings (Barber et al., 2009; Andretta et al., 2014; King, 2016; Crone et al., 2020; Mazzetti et al., 2020; Coughlin et al., 2022), a broad consensus as to the functional benefit of future-oriented thought within academic settings has yet to be reached. In other words, to date there had been no effort to combine and contrast findings in this field with the sole intention of reaching such a conclusion. To address the central aim, we critically reviewed the literature and identified studies for inclusion into the review. Only studies that emphasized academic outcomes were included in the review. Through a stringent inclusion protocol, 21 high-quality studies were included, and data was extracted from each study. After reviewing 21 independent studies, our primary finding is that the degree to which an individual is future-oriented has direct implications on their levels of engagement, and overall performance within an academic setting. Building on a previous meta-analysis by Kooij et al. (2018), the current study extends these findings by assessing the efficacy of future-oriented thought in promoting positive outcomes in academia whilst considering conditions in which future-oriented thought is proven to be more beneficial.

### Future-oriented thought: inventories and academic outcomes

Ten independent studies were included to assess whether individual levels of future-oriented are correlated to positive academic outcomes. Consequently, it was identified that those who have higher levels of future orientation demonstrated greater levels of classroom attendance, engagement, GPA, and hours of study completed. This suggests that individuals who are oriented toward the future are likely to demonstrate positive outcomes in academic settings.

#### Future-oriented thought: interventions and academic outcomes

Eleven independent studies were included to assess whether the application of inventories found to increase levels of future orientation were also successful in increasing positive academic outcomes. Findings indicate that the utility of interventions to increase the degree to which one is future-oriented yields positive outcomes in academic settings. Broadly speaking, as an individual's orientation toward the future increases, so too does their academic performance.

#### Overall effects of future-oriented thought on academic outcomes

With regard to the overall effect of future-oriented thought on academic outcomes, the findings of our systematic review support the use of future-oriented thought practices within academic settings in order to promote positive outcomes. This position is supported through consistent evidence identified collectively, and at intervention and inventory group levels demonstrating the positive outcomes discussed. The overall effect in the present study indicates that when considering desired outcomes in academic environments, such as primary school, high-school, as well as tertiary settings, specifically in relation to engagement (class attendance; contribution; homework completion; and study hours) as well as performance (overall Grade Point Average) collectively, greater future orientation is indicative of positive outcomes in academic settings. The function that future-oriented thought serves from a behavioral perspective can be argued to be related to increases in effective decision making and interpersonal organization. In other words, when increases of future orientation were identified, so too were levels of classroom engagement as well as increases in hours of study. Previous research conducted by De Beni and Moè (2003) exploring imagery and rehearsal suggest that this perspective is plausible.

The current results provide further empirical support for future-oriented thought as a mechanism for facilitating behaviors congruent with desired outcomes. The current systematic review also considered the varying outcomes each study investigated which formed subgroups within our systematic review, performance related (GPA) and outcomes related to engagement; critically informing whether the degree to which one is more future-oriented is indicative of desirable outcomes such as performance, or engagement. In contrast to previous meta-analysis studies (Andre et al., 2018; Kooij et al., 2018; Loveday et al., 2018), the current study provides an important contribution to the understanding as to the benefit of future-oriented thought in promoting positive outcomes within an academic setting. Our intentions of exploring future-oriented thought in academic settings was to elucidate the adaptive functions of future-oriented thought in eliciting behaviors congruent with desired outcomes in academic contexts, such as outcomes related to engagement. Facilitating future-oriented thought has the potential to increase student capabilities to succeed in their chosen degree. By doing so, we extended the theoretical understanding of the ability for future-oriented thought to promote positive outcomes.

## Conclusion

It was posited at the beginning of this study that future-oriented thought has been identified to be related to broadly positive outcomes more in everyday life, and specifically within academic contexts. However, there are no prior investigations identifying whether future-oriented thought is beneficial in academic settings. In the present study, we found evidence that suggests future-oriented thought is indeed beneficial when seeking to enhance student engagement and performance within the academic environment. With regard to engagement, we found that those who demonstrated greater future orientation displayed greater levels of engagement when compared to baseline measures. With regard to performance, we found that those who are more future-oriented displayed greater GPA when compared against baseline measures. In conclusion, the present study offers important insights when considering strategies that may increase the level of student engagement and performance within academia.

### Implications

We can conclude from this systematic review is the efficacy of future-oriented thought in an academic setting, as evidenced by the positive outcomes discussed, such as, student engagement and improvements in GPA. The variety of future-oriented thought interventions presented above (see future-oriented thought: inventories and interventions) can be pragmatically utilized to form a robust approach to improving academic outcomes of students at risk of failure. In order to increase the degree to which one is more future-oriented in the classroom, the interventions discussed in this review, such as mental contrasting with implementation intentions (Kirk et al., 2012), or possible selves (Oyserman and James, 2012), were shown to be useful in pragmatically implementing on either at individual or group levels. Education providers that wish to take a more engaged approach to improve academic outcomes of students at risk of failure may consider this systematic review as a case study. With respect to the findings of this review, contrasting a chosen academic path to the way in which one constructs their future may lead to an increase in classroom attendance, engagement, participation, as well as improvements in GPA.

### Limitations and future directions

This study is not without limitations. Firstly, statistical heterogeneity is one of the primary threats to meta-analyses, however, as noted by Higgins (2008) it is to be expected when dealing with differences in data. This was specifically evident in studies that assessed similar outcomes utilizing different future-oriented measures. However, all reasonable steps were taken in this instance to ensure heterogeneity was assessed appropriately. Secondly, although the number of included studies in this systematic review was limited to 20, several studies were not able to be included due to an inability to contact authors to discuss information about these studies.

An interesting, unexplored, and potentially meaningful line of inquiry that has been identified upon the completion of the current systematic review is the utility of future-oriented thought in undergraduate degrees, and specifically, as an intervention for undergraduate students who have been identified to be at risk of failure or who are stagnating in performance. Given the identified efficacy of future-oriented thought in this study, it would be beneficial to explore how these populations perceive their future, and whether increasing the degree to which one is future-oriented is indicative of increased levels of motivation, in-turn lifting student engagement.

## Author contributions

All authors listed have made a substantial, direct, and intellectual contribution to the work and approved it for publication.

## References

[B1] Adelabu D. H. (2008). Future Time Perspective, Hope, and Ethnic Identity Among African American Adolescents. Urban Educ. 43, 347–360. 10.1177/0042085907311806

[B2] Adelman R. M. Herrmann S. D. Bodford J. E. Barbour J. E. Graudejus O. Okun M. A. . (2017). Feeling closer to the future self and doing better: temporal psychological mechanisms underlying academic performance. J. Pers. 85, 398–408. 10.1111/jopy.12248 26900025

[B3] Anderson R. J. Evans G. L. (2015). Mental time travel in dysphoria: differences in the content and subjective experience of past and future episodes. Consci. Cognition 37, 237–248. 10.1016/j.concog.2014.05.006 25066660

[B4] Andre L. van Vianen A. E. Peetsma T. T. Oort F. J. (2018). Motivational power of future time perspective: meta-analyses in education, work, and health. PLoS ONE, 13, 492. 10.1371/journal.pone.0190492 29364917 PMC5783357

[B5] Andretta J. R. Worrell F. C. Mello Z. R. (2014). Predicting educational outcomes and psychological wellbeing in adolescents using time attitude profiles. Psychology in the Schools, 51, 434–451. 10.1002/pits.21762

[B6] Arnett J. J. Žukauskiene R. Sugimura K. (2014). The new life stage of emerging adulthood at ages 18–29 years: Implications for mental health. Lancet Psychiatry 1, 569–576. 10.1016/S2215-0366(14)00080-7 26361316

[B7] Atance C. M. O'Neill D. K. (2005). The emergence of episodic future thinking in humans. Learn. Motivat. 36, 126–144. 10.1016/j.lmot.2005.02.003

[B8] Barber L. K. Munz D. C. Bagsby P. G. Grawitch M. J. (2009). When does time perspective matter? Self-control as a moderator between time perspective and academic achievement. Pers. Individ. Dif. 46, 250–253. 10.1016/j.paid.2008.10.007

[B9] Barnett M. D. Hernandez J. Melugin P. R. (2019). Influence of future possible selves on outcome expectancies, intended behaviour, and academic performance. Psychol. Rep. 122, 2320–2330. 10.1177/0033294118806483 30426837

[B10] Barnett M. D. Melugin P. R. Hernandez J. (2020). Time perspective, intended academic engagement, and academic performance. Curr. Psychol, 39, 761–767. 10.1007/s12144-017-9771-9

[B11] Bartels D. M. Urminsky O. (2011). On intertemporal selfishness: How the perceived instability of identity underlies impatient consumption. J. Consum. Res. 38, 182–198. 10.1086/658339

[B12] Berkman E. T. (2018). The neuroscience of goals and behaviour change. Consul. Psychol. J. Pract. Res. 70, 28. 10.1037/cpb0000094 29551879 PMC5854216

[B13] Bowles T. (2008). The relationship of time orientation with perceived academic performance and preparation for assessment in adolescents. Edu. Psychol. 28, 551–565. 10.1080/01443410701880134

[B14] Bulley A. Henry J. Suddendorf T. (2016). Prospection and the present moment: The role of episodic foresight in intertemporal choices between immediate and delayed rewards. Rev. Gen. Psychol. 20, 29–47. 10.1037/gpr0000061

[B15] Carraro N. Gaudreau P. (2011). Implementation planning as a pathway between goal motivation and goal progress for academic and physical activity goals. J. App. Soc. Psychol. 41, 1835–1856.

[B16] Chishima Y. Wilson A. E. (2021). Conversation with a future self: a letter-exchange exercise enhances student self-continuity, career planning, and academic thinking. Self Identity,20, 646–671. 10.1080/15298868.2020.1754283

[B17] Clark M. Miller A. Berry J. Cheng K. (2021). Mental contrasting with implementation intentions increases study time for university students. Br. J. Educ. Psychol. 91, 850–864. 10.1111/bjep.12396 33315247

[B18] Coughlin C. Prabhakar J. D'Esposito Z. Thigpen B. Ghetti S. (2022). Promoting future-oriented thought in an academic context. Cogn. Dev. 62, 101183. 10.1016/j.cogdev.2022.101183

[B19] Crone L. Brunel L. Auzoult L. (2020). Can temporal anticipation of the “academic success” event influence actual performance? Pychol. Edu. 57, 1089–1095.

[B20] Cross A. Sheffield D. (2019). Mental contrasting for health behaviour change: a systematic review and meta-analysis of effects and moderator variables. Health Psychol. Rev. 13, 209–225. 10.1080/17437199.2019.1594332 30879403

[B21] De Beni and Moè, R.. (2003). Imagery and rehearsal as study strategies for written or orally presented passages. Psychonomic Bullet. Review 10, 975–980. 10.3758/BF03196561 15000548

[B22] de Place and Brunot, A. L.. (2020). Motivational and behavioral impact of possible selves: when specificity matters. Imagin. Cogn. Pers. 39, 329–347. 10.1177/0276236619864275

[B23] Delfino A. P. (2019). Student engagement and academic performance of students of Partido State University. Asian J. Univ. Education, 15, 22–41. 10.24191/ajue.v15i3.05

[B24] Dogan U. (2015). Student engagement, academic self-efficacy, and academic motivation as predictors of academic performance. The Anthropologis, 20, 553–561. 10.1080/09720073.2015.11891759

[B25] Duckworth A. L. Kirby T. A. Gollwitzer A. Oettingen G. (2013). From fantasy to action: mental contrasting with implementation intentions (MCII) improves academic performance in children. Soc. Psychol. Personal. Sci. 4, 745–753. 10.1177/1948550613476307 25068007 PMC4106484

[B26] Engin G. (2020). An examination of primary school students' academic achievements and motivation in terms of parents' attitudes, teacher motivation, teacher self-efficacy and leadership approach. Int. J. Prog. Edu. 16, 257–276. 10.29329/ijpe.2020.228.18

[B27] Ernst A. Philippe F. L. d'Argembeau A. (2018). Wanting or having to: The role of goal self-concordance in episodic future thinking. Consc. Cogn. 66, 26–39. 10.1016/j.concog.2018.10.004 30391628

[B28] Ewert A. Sibthorp J. (2009). Creating outcomes through experiential education: the challenge of confounding variables. J. Exp. Edu. 31, 376–389. 10.1177/105382590803100305

[B29] Foster A. Shah M. Barany A. Talafian H. (2019). *High school students' role-playing for identity* exploration: findings from virtual city planning. Inform. Learn. Sci. 3, 26. 10.1108/ILS-03-2019-0026

[B30] Gerber C. Mans-Kemp N. Schlechter A. (2013). Investigating the moderating effect of student engagement on academic performance. Acta Acad. 45, 256–274.

[B31] Gjesme T. (1975). Slope of gradients for performance as a function of achievement motive, goal distance in time, and future time orientation. J. Psychol. 91, 143–160. 1206608 10.1080/00223980.1975.9915808

[B32] Gollwitzer A. Oettingen G. Kirby T. A. Duckworth A. L. Mayer D. (2011). Mental contrasting facilitates academic performance in school children. Motiv. Emot. 35, 403–412. 10.1007/s11031-011-9222-0

[B33] Gupta M. Bakker A. B. (2020). *Future time perspective and group performance among* students: role of student engagement and group cohesion. J. App. Res. Higher Edu. 3, 128 10.1108/JARHE-05-2019-0128

[B34] Gupta R. Hershey D. A. Gaur J. (2012). Time Perspective and Procrastination in the Workplace: an Empirical Investigation. Curr. Psychol. 31, 195–211. 10.1007/s12144-012-9136-3

[B35] Hagenauer G. Hascher T. Volet S. E. (2015). Teacher emotions in the classroom: associations with students' engagement, classroom discipline and the interpersonal teacher-student relationship. Eur. J. Psychol. Edu. 30, 385–403. 10.1007/s10212-015-0250-0

[B36] Henry A. (2020). Possible selves and personal goals: what can we learn from episodic future thinking? Eurasian J. Applied Ling. 6, 481–500. 10.32601/ejal.834659

[B37] Higgins J. P. (2008). Commentary: heterogeneity in meta-analysis should be expected and appropriately quantified. Int. J. Epidemiol. 37, 1158–1160. 10.1093/ije/dyn204 18832388

[B38] Hollis-Hansen K. O'Donnell S. E. Seidman J. S. Brande S. J. Epstein L. H. (2019). Improvements in episodic future thinking methodology: Establishing a standardized episodic thinking control. PLoS ONE, 14, e0214397. 10.1371/journal.pone.0214397 30921384 PMC6438451

[B39] Horstmanshof L. Zimitat C. (2007). Future time orientation predicts academic engagement among first-year university students. British Journal of Educational Psychology, 77, 703–718. 10.1348/000709906X160778 17908382

[B40] Hoyle R. H. Sherrill M. R. (2006). Future orientation in the self-system: Possible selves, self-regulation, and behaviour. J. Pers., 74, 1673–1696. 10.1111/j.1467-6494.2006.00424.x 17083662

[B41] Husman J. Shell D. F. (2008). Beliefs and perceptions about the future: A measurement of future time perspective. Learn. Individ. Differ., 18, 166–175. 10.1016/j.lindif.2007.08.001

[B42] Kappes H. B. Oettingen G. Mayer D. (2012). Positive fantasies predict low academic achievement in disadvantaged students. Eur. J. Soc. Psychol., 42, 53–64. 10.1002/ejsp.838

[B43] Kim L. E. Dar-Nimrod I. MacCann C. (2018). Teacher Personality and Teacher Effectiveness in Secondary School: Personality Predicts Teacher Support and Student Self-Efficacy but Not Academic Achievement. Journal of Educational Psychology, 110, 309–323. 10.1037/edu0000217

[B44] King L. A. (2001). The health benefits of writing about life goals. Pers. Soc. Psychol. Bull. 27, 798–807. 10.1177/0146167201277003

[B45] King R. B. (2016). Does your approach to time matter for your learning? The role of time perspectives on engagement and achievement. Educational Psychology, 36, 1264–1284. 10.1080/01443410.2015.1045835

[B46] Kinik Ö. Odaci H. (2020). Effects of dysfunctional attitudes and depression on academic procrastination: does self-esteem have a mediating role?. British Journal of Guidance and Counselling, 48, 638–649. 10.1080/03069885.2020.1780564

[B47] Kirk D. Oettingen G. Gollwitzer P. M. (2012). Promoting Integrative Bargaining: Mental Contrasting with Implementation Intentions. New York: Psychology Department. New York University; New York, New York. 10.1108/10444061311316771

[B48] Kooij D. T. Kanfer R. Betts M. Rudolph C. W. (2018). Future time perspective: a systematic review and meta-analysis. J. Appl. Psychol. 103, 867–893. 10.1037/apl0000306 29683685

[B49] Koul R. Sosik J. J. Lerdpornkulrat T. (2017). Students' possible selves and achievement goals: examining personal and situational influences in Thailand. Sch. Psychol. Int. 38, 408–433. 10.1177/0143034317702946

[B50] Lamb S. Huo S. Walstab A. Wade A. Maire Q. Doecke E. . (2020). Educational Opportunity in Australia 2020: Who Succeeds and Who Misses Out. Melbourne: Centre for International Research on Education Systems, Victoria University, for the Mitchell Institute.

[B51] Landau M. J. Barrera J. Keefer L. A. (2017). On the road: combining possible identities and metaphor to motivate disadvantaged middle-school students. Metaphor Symb. 34, 276–290. 10.1080/10926488.2017.1384271

[B52] Landau M. J. Oyserman D. Keefer L. A. Smith G. C. (2014). The college journey and academic engagement: how metaphor use enhances identity-based motivation. J. Personal. Soc. Psychol. 106, 679. 10.1037/a0036414 24749818

[B53] Lewin K. (1942). “Time perspective and morale.” in Resolving social conflicts ed G. Lewin (pp. 103–124). New York: Harper. 10.1037/13983-004

[B54] Li L. Yang S. (2021). Exploring the influence of teacher-student interaction on university students' self-efficacy in the flipped classroom. J. Edu. Learn. 10, 84–90. 10.5539/jel.v10n2p84

[B55] Loveday P. M. Lovell G. P. Jones C. M. (2018). The best possible selves intervention: a review of the literature to evaluate efficacy and guide future research. J. Happiness Stud. 19, 607–628.

[B56] Madrazo F. J. P. (2021). Learning Attitude as Predictor on the Academic Performance of Grade V Pupils in Science. Delhi: International journal of advanced multidisciplinary studies.

[B57] Markus and Nurius, P.. (1986). Possible selves. Am. Psychol. 41, 954–969. 10.1037/0003-066X.41.9.954

[B58] Mazzetti G. Paolucci A. Guglielmi D. Vannini I. (2020). The impact of learning strategies and future orientation on academic success: the moderating role of academic self-efficacy among Italian undergraduate students. Edu. Sci. 10, 134. 10.3390/educsci10050134

[B59] McGuire W. (1990). Dynamic operations of thought systems. Am. Psychol. 45, 504–512. 10.1037/0003-066X.45.4.504 2337290

[B60] McMichael S. L. Bixter M. T. Okun M. A. Bunker C. J. Graudejus O. Grimm K. J. . (2022). Is seeing believing? A longitudinal study of vividness of the future and its effects on academic self-efficacy and success in college. Personal. Soc. Psychol. Bulletin 48, 478–492. 10.1177/01461672211015888 34018855

[B61] Mello Z. R. Worrell F. C. (2006). The relationship of time perspective to age, gender, and academic achievement among academically talented adolescents. J. Edu. Gifted 29, 271–289. 10.1177/016235320602900302

[B62] Mello Z. R. Worrell F. C. Buhl M. (2008). The Adolescent Time Inventory–German. German Institute for International Educational Research, University of Frankfurt/Main, and the University of California, Berkeley. Available online at: http://www.uccs.edu/zmello/ati.~html.

[B63] Miller R. B. Brickman S. J. (2004). A model of future-oriented motivation and self-regulation. Educ. Psychol. Rev. 16, 9–33. 10.1023/B:EDPR.0000012343.96370.39

[B64] Moher D. Liberati A. Tetzlaff J. Altman D. G. Group P. R. I. S. M. A. (2009). Preferred reporting items for systematic reviews and meta-analyses: the PRISMA statement. Ann. Intern. Med. 151, 264–269. 10.7326/0003-4819-151-4-200908180-00135 19622511

[B65] Moustafa A. A. Morris A. N. ElHaj, M. (2019). A review on future episodic thinking in mood and anxiety disorders. Rev. Neurosci. 30, 85–94. 10.1515/revneuro-2017-0055 29858910

[B66] Moustafa A. A. Morris A. N. Nandrino J. L. Misiak B. Szewczuk-Bogusławska M. Frydecka D. . (2018). Not all drugs are created equal: impaired future thinking in opiate, but not alcohol users. Exp. Brain Res. 236, 2971–2981. 10.1007/s00221-018-5355-7 30099573

[B67] Nurmi J. E. (1991). How do adolescents see their future? A review of the development of future orientation and planning. Develop. Rev. 11, 1–59. 10.1016/0273-2297(91)90002-627409075

[B68] O'Donnell S. Daniel T. O. Epstein L. H. (2017). Does goal relevant episodic future thinking amplify the effect on delay discounting? Conscious. Cogn. 51, 10–16. 10.1016/j.concog.2017.02.014 28282631 PMC5651988

[B69] Oettinger G. Mayer D. (2002). The motivating function of thinking about the future: epectations vs. fantasies. J. Personal. Soc. Psychol. 83, 1198–1212. 10.1037/0022-3514.83.5.1198 12416922

[B70] Oyserman D. Bybee D. Terry K. (2006). Possible selves and academic outcomes: how and when possible selves impel action. J. Pers. Soc. Psychol., 91, 188. 10.1037/0022-3514.91.1.188 16834488

[B71] Oyserman D. Destin M. Novin S. (2015). The context-sensitive future self: possible selves motivate in context, not otherwise. Self Ident. 14, 173–188. 10.1080/15298868.2014.965733

[B72] Oyserman D. James L. (2012). “Possible selves: from content to process,” in Handbook of Imagination and Mental Simulation. Psychology Press (pp. 373-394).

[B73] Peetz J. Wilson A. E. Strahan E. J. (2009). So far away: the role of subjective temporal distance to future goals in motivation and behavior. Soc. Cogn. 27, 475. 10.1521/soco.2009.27.4.475

[B74] Peters B. R. Joireman J. Ridgway R. L. (2005). Individual differences in the consideration of future consequences scale correlate with sleep habits, sleep quality, and GPA in University students. Psychol. Reports 96, 817–824. 10.2466/pr0.96.3.817-824 16050645

[B75] Rieckmann M. (2012). Future-oriented higher education: which key competencies should be fostered through university teaching and learning? Futures 44, 127–135. 10.1016/j.futures.2011.09.005

[B76] Rissanen A. (2018). Student engagement in large classroom: the effect on grades, attendance and student experiences in an undergraduate biology course. Canad. J. Sci. Math. Technol. Educ. 18, 136–153. 10.1007/s42330-018-0015-2

[B77] Robbins S. B. Lauver K. Le H. Davis D. Langley R. Carlstrom A. (2004). Do psychosocial and study skill factors predict college outcomes? A meta-analysis. Psychol. Bull. 130, 261–288. 10.1037/0033-2909.130.2.261 14979772

[B78] Ruvolo A. P. Markus H. R. (1992). Possible selves and performance: the power of self-relevant imagery. Soc. Cogn. 10, 95–124. 10.1521/soco.1992.10.1.95

[B79] Schacter D. L. Addis D. R. Buckner R. L. (2008). Episodic simulation of future events: concepts, data, and applications. Ann. New York Acad. Sci. 1124, 39–60. 10.1196/annals.1440.001 18400923

[B80] Schacter D. L. Benoit R. G. Szpunar K. K. (2017). Episodic future thinking: mechanisms and functions. Curr. Opin. Behav. Sci. 17, 41–50. 10.1016/j.cobeha.2017.06.002 29130061 PMC5675579

[B81] Shell D. F. Husman J. (2001). The multivariate dimensionality of personal control and future time perspective beliefs in achievement and self-regulation. Contemp. Edu. Psychol. 26, 481–506. 10.1006/ceps.2000.1073 11681829

[B82] Shipley B. Ian W. (2019). Here comes the Drop: *University Drop-Out Rates and Increasing Student Retention Through Education*. Australia: National Centre for Vocational Education Research.

[B83] Shulman S. Barr T. Livneh Y. Nurmi J. E. Vasalampi K. Pratt M. (2015). Career pursuit pathways among emerging adult men and women: Psychosocial correlates and precursors. Int. J. Behav. Develop. 39, 9–19. 10.1177/0165025414533222

[B84] Stanescu D. F. Iorga E. M. (2015). An exploratory study regarding the relations between time perspective, achievement motivation and self-regulation. Manag. Dyn. Knowledge Econ. 3, 7.27409075

[B85] Stein J. S. Tegge A. N. Turner J. K. Bickel W. K. (2018). Episodic future thinking reduces delay discounting and cigarette demand: an investigation of the good-subject effect. J. Behav. Med. 41, 269–276. 10.1007/s10865-017-9908-1 29270887

[B86] Strathman A. Gleicher F. Boninger D. S. Edwards C. S. (1994). The consideration of future consequences: weighing immediate and distant outcomes of behaviour. J. Pers. Soc. Psychol., 66, 742. 10.1037/0022-3514.66.4.742

[B87] Study Australia (2022). Higher Education Qualifications. Australian Government, Universities and Higher Education. Avilalbe online at: https://www.studyaustralia.gov.au/english/study/universities-higher-education/higher-education-qualifications

[B88] Szpunar K. K. McDermott K. B. (2008). Episodic future thought: Remembering the past to imagine the future. In Markman, K.D., Klein, W.M.P., Suhr, J.A. (Eds.), The handbook of imagination and mental simulation (pp. 119–129). New York: Psychology Press.

[B89] Szpunar K. K. Schacter D. L. (2013). Get real: effects of repeated simulation and emotion on the perceived plausibility of future experiences. J. Exp. Psychol. General 142, 323. 10.1037/a0028877 22686637 PMC3461111

[B90] Szpunar K. K. Spreng R. N. Schacter D. L. (2014). A taxonomy of prospection: Introducing an organizational framework for future-oriented cognition. Proc. Nat. Acad. Sci., 111, 18414–18421. 10.1073/pnas.1417144111 25416592 PMC4284580

[B91] Tindle R. Hamza E. G. A. Helal A. A. Ayoub A. E. A. Moustafa A. (2021). A Systematic Review of the Psychosocial Correlates of Academic Performance. 10.31234/osf.io/frscp22352812

[B92] Worrell F. C. Perry J. L. Wells K. E. McKay M. T. (2021). Time to change your attitude? Socio-economic status, academic attainment, and time attitudes in Glasgow school children. Int. J. School Edu. Psychol. 9, 280–289. 10.1080/21683603.2020.1856740

[B93] Zimbardo P. G. Boyd J. N. (1999). Putting time in perspective: a valid, reliable individual-differences metric. J. Pers. Soc. Psychol. 77, 1271–1288. 10.1037/0022-3514.77.6.1271

